# Small molecules facilitate single factor-mediated sweat gland cell reprogramming

**DOI:** 10.1186/s40779-022-00372-5

**Published:** 2022-03-29

**Authors:** Shuai-Fei Ji, Lai-Xian Zhou, Zhi-Feng Sun, Jiang-Bing Xiang, Shao-Yuan Cui, Yan Li, Hua-Ting Chen, Yi-Qiong Liu, Huan-Huan Gao, Xiao-Bing Fu, Xiao-Yan Sun

**Affiliations:** 1 Research Center for Tissue Repair and Regeneration Affiliated To Medical Innovation Research Department and 4th Medical Center, PLA General Hospital and PLA Medical College; PLA Key Laboratory of Tissue Repair and Regenerative Medicine and Beijing Key Research Laboratory of Skin Injury, Repair and Regeneration, 28 Fu Xing Road, Beijing, 100853 China; 2 Research Unit of Trauma Care, Tissue Repair and Regeneration, Chinese Academy of Medical Sciences, 2019RU051, Beijing, 100048 China; 3 Department of Respiratory, The Second Medical Center, Chinese PLA General Hospital, Beijing, 100036 China; 4 Bioengineering College of Chongqing University, Chongqing, 400044 China; 5 Department of Nephrology, The First Medical Center, Chinese PLA General Hospital, State Key Laboratory of Kidney Diseases, Beijing, 100048 China

**Keywords:** Direct reprogramming, Human dermal fibroblasts, Sweat gland, Regeneration

## Abstract

**Background:**

Large skin defects severely disrupt the overall skin structure and can irreversibly damage sweat glands (SG), thus impairing the skin’s physiological function. This study aims to develop a stepwise reprogramming strategy to convert fibroblasts into SG lineages, which may provide a promising method to obtain desirable cell types for the functional repair and regeneration of damaged skin.

**Methods:**

The expression of the SG markers cytokeratin 5 (CK5), cytokeratin 10 (CK10), cytokeratin 18 (CK18), carcino-embryonic antigen (CEA), aquaporin 5 (AQP5) and α-smooth muscle actin (α-SMA) was assessed with quantitative PCR (qPCR), immunofluorescence and flow cytometry. Calcium activity analysis was conducted to test the function of induced SG-like cells (iSGCs). Mouse xenograft models were also used to evaluate the in vivo regeneration of iSGCs. BALB/c nude mice were randomly divided into a normal group, SGM treatment group and iSGC transplantation group. Immunocytochemical analyses and starch-iodine sweat tests were used to confirm the in vivo regeneration of iSGCs.

**Results:**

EDA overexpression drove HDF conversion into iSGCs in SG culture medium (SGM). qPCR indicated significantly increased mRNA levels of the SG markers CK5, CK18 and CEA in iSGCs, and flow cytometry data demonstrated (4.18 ± 0.04)% of iSGCs were CK5 positive and (4.36 ± 0.25)% of iSGCs were CK18 positive. The addition of chemical cocktails greatly accelerated the SG fate program. qPCR results revealed significantly increased mRNA expression of CK5, CK18 and CEA in iSGCs, as well as activation of the duct marker CK10 and luminal functional marker AQP5. Flow cytometry indicated, after the treatment of chemical cocktails, (23.05 ± 2.49)% of iSGCs expressed CK5^+^ and (55.79 ± 3.18)% of iSGCs expressed CK18^+^, respectively. Calcium activity analysis indicated that the reactivity of iSGCs to acetylcholine was close to that of primary SG cells [(60.79 ± 7.71)% vs. (70.59 ± 0.34)%, ns]. In vivo transplantation experiments showed approximately (5.2 ± 1.1)% of the mice were sweat test positive, and the histological analysis results indicated that regenerated SG structures were present in iSGCs-treated mice.

**Conclusion:**

We developed a SG reprogramming strategy to generate functional iSGCs from HDFs by using the single factor EDA in combination with SGM and small molecules. The generation of iSGCs has important implications for future in situ skin regeneration with SG restoration.

**Supplementary Information:**

The online version contains supplementary material available at 10.1186/s40779-022-00372-5.

## Background

Sweat glands (SGs), which are found over almost the entire body, have important functions in maintaining temperature homeostasis through secretion of sweat in response to heat and exercise [1, 2]. However, large skin defects caused by burns, severe trauma or chronic cutaneous wounds can lead to disruption of the overall structure and physiological function of the skin, as well as irreversible damage to SGs. SG damage is followed by heat intolerance and thermoregulatory dysfunction, thus threatening health and quality of life. Despite great progress in clinical management for skin wounds, restoration of damaged SGs is far from possible; thus, functional repair of skin wounds with regenerated SGs remains an essential and challenging issue.

Endogenous stem cell populations have been reported in SGs during embryonic development. However, lineage-tracing experiments have indicated that these precursor/stem cells have limited self-renewal and regeneration potential, exhibit limited responses to tissue injury stress and do not form SGs during wound healing [3]. To overcome these challenges, cellular reprogramming offers an attractive novel strategy for the acquisition of high-quality SG cells on a large scale for the functional repair and regeneration of damaged skin tissues. Mesenchymal stromal cells and epidermal keratinocytes can be converted to induced SG cells (iSGCs) through the modulation of ectodermal dysplasia antigen (EDA) expression [4, 5]. The reprogrammed cells not only express the SG cell markers cytokeratin (CK)5, CK10, CK18, CK19, CK14, CEA and aquaporin 5 (AQP5), but also facilitate the restoration of SGs in vivo. The enforced expression of lineage-specific factors has been extensively used in the reprogramming strategy [6, 7]; however, no single factor has been sufficient for cellular reprogramming [8, 9]. Combinatorial approaches for direct lineage conversion must be constructed [10]. Small molecules, through non-viral and non-integrating approaches, have been shown to enhance single factor-mediated reprogramming efficiency [11, 12]; such molecules include Repsox (an ALK4/5/7 inhibitor), CHIR99021 (a GSK3β inhibitor), parnate (an LSD1/KDM1 inhibitor, also called tranylcypromine), TTNPB [a retinoic acid (RA) receptor ligand] and forskolin (an adenylyl cyclase activator) [13, 14].

Fibroblasts have been extensively used as the starting cells in most direct reprogramming experiments, owing to their availability and potential for in vivo reprogramming [15–17]. In particular, dermal fibroblasts are a dominant cell type involved in cutaneous wound repair and regeneration [18]. Accordingly, the reprogramming of dermal fibroblasts is important for in-situ repair and regeneration of damaged skin. On the basis of prior studies, we hypothesized that SG cells might be generated directly from dermal fibroblasts via the combination of EDA overexpression and small molecules.

In the present study, we aimed to develop a stepwise strategy involving EDA overexpression in combination with defined small-molecule cocktails to promote the transition of human dermal fibroblasts (HDFs) into functional iSGCs. We expected that EDA manipulation would direct HDFs toward the SG fate, and that SG culture medium (SGM) culture with HDFs transfected with an EDA sequence would acquire SG cell identity. Finally, we hypothesized that the chemical cocktails would accelerate the reprogramming of HDFs to iSGCs.

## Methods

The principal purpose of this research was to develop a direct reprogramming strategy using single factor EDA in combination with small-molecule cocktails to promote cell-fate conversion and regenerate SG cells from HDFs. Quantitative PCR (qPCR), immunofluorescence and flow cytometry were used to assess the phenotypes of HDF-derived iSGCs and analyze the reprogramming efficiency. Calcium activity analysis was conducted to evaluate the in vitro function of HDF-derived iSGCs. A total of 188 BALB/c nude mice were randomly divided into a normal group, modified SGM treatment group and iSGC transplantation group. Immunocytochemical analyses and starch-iodine sweat tests were used to characterize the in vivo regeneration of SG cells.

### Isolation and culture of HDFs and primary SG cells (pSGCs)

HDFs were isolated from foreskin specimens from 15–19 years old males. The skin tissue was washed with phosphate-buffered saline (PBS) containing penicillin (100 U/ml) and streptomycin (100 μg/ml). After washing, the skin tissue was cut into small pieces (1 – 2 mm^3^) and placed in a Petri dish with fibroblast medium comprising high glucose DMEM (Gibco, USA) and 10% fetal bovine serum (Gibco). The culture medium was washed and digested with 0.25% trypsin–EDTA for cell passage, and the subsequent experiments were performed after five to nine fibroblast passages. The pSGCs were isolated from normal breast tissue. The present studies were approved by the Clinical Research Ethics Committee of General Hospital of PLA (Beijing, China), and written informed consent was obtained from all individuals before samples were obtained.

### Generation of iSGCs

HDFs between passages five and nine were used for iSGCs generation. The viral particles for iSGCs generation were produced after transfection of 293FT cells with a single pMX retroviral vector encoding EDA (pLV-hef1a-EDA-GFP) together with packaging plasmid psPAX2 and envelope plasmid pMD2.G. Lipofectamine™ 2000 was used for further transfection according to the manufacturer’s instructions. Puromycin with a final concentration of 0.4 µg/ml was added at 48 h post-transfection to obtain stably transfected cells, and puromycin selection was continued for 15 d. HDFs transfected with EDA were cultured in SGM in the presence or absence of the following small molecules and proteins: Repsox (10 μmol/L, Selleck, USA), CHIR99021 (10 μmol/L, Selleck, USA), isoproterenol (5 μmol/L, Sigma-Aldrich, USA), RA (10 μmol/L, Sigma-Aldrich, USA) and BMP4 (20 ng/ml, R&D, USA). SGM consisted of DMEM/F12 medium (Gibco) supplemented with 10% fetal bovine serum (Gibco), 1 × B27 (17,504,044, Gibco), 1 × Glutamax™ (Gibco), human epidermal growth factor (50 ng/ml, Sigma-Aldrich), basic fibroblast growth factor (20 ng/ml, R&D), penicillin (100 U/ml) and streptomycin (100 μg/ml).

### RNA extraction and qPCR

Total RNA was extracted from cells with TRIzol reagent (Invitrogen, USA), as recommended by the manufacturer. cDNA was generated by reverse transcription of total RNA (500 ng) with a PrimeScript RT reagent kit (TaKaRa, Japan). qPCR was performed, and SYBR Green Supermix (Bio-Rad, USA) was used for relative quantification of the indicated genes. Quantification of target genes was normalized against the input on the basis of β-actin. The primer sequences for qPCR are listed in Additional file 1: Table S1.

### Immunofluorescence and immunohistochemistry

For immunofluorescence staining, cells were fixed in 4% paraformaldehyde (Solarbio, China) at room temperature for 30 min and permeabilized in 0.2% Triton X-100 in PBS (PBST) for 10 min. The cells were then blocked with PBST containing 5% normal goat serum (Solarbio) at room temperature for 30 min. Cells were incubated with primary antibodies overnight at 4℃ and secondary antibodies at room temperature for 2 h. The primary antibodies used in this study were rabbit anti-CK5 (ab52635, 1:200, Abcam,USA), mouse anti-α-SMA (ab7817, 1:200, Abcam, USA), rabbit anti-CK18 (ab133263, 1:200, Abcam) and rabbit Alexa Fluor® 647 anti-aquaporin 5 (ab215225, 1:200, Abcam, USA). The following secondary antibodies were used: goat anti-rabbit IgG H&L (Alexa Fluor 594) (ab150080, 1:200, Abcam, USA) and goat anti-mouse IgG H&L (Alexa Fluor 647) (ab150115, 1:200, Abcam, USA).

For immunohistochemistry staining, antigen retrieval of samples was performed in 10 mmol/L citric acid buffer (pH 6.0) for 15 min, and 0.3% H_2_O_2_ was added to block endogenous peroxidase activity. Then slides were incubated with rabbit anti-CK5 (ab52635, 1:200, Abcam, USA), rabbit anti-CK18 (ab133263, 1:200, Abcam, USA) and rabbit anti-CK14 (ab119695, 1:200, Abcam, USA) overnight at 4℃. Antibody binding was detected with a streptavidin–biotin-peroxidase immunohistochemical system (SP-9000, ZSGB-BIO), and color development was detected through DAB staining (ZLI-9017, ZSGB-BIO). The slides were counterstained with hematoxylin.

### FACS analysis

For detection of intracellular antigens, single cells were fixed with 4% paraformaldehyde and permeabilized with PBST for 10 min. Cells were then blocked with 5% goat serum and incubated with flow cytometry antibodies, including APC-conjugated CK5 (ab224984, 1:100, Abcam, USA) and PE-conjugated CK18 (ab210410, 1:100, Abcam, USA). A FACStar Plus Flow Cytometer (BD Biosciences, USA) was used for further analysis and quantification of FACS data.

### Calcium activity analysis

For calcium activity analysis, HDFs, HDF-EDA cells treated with SGM, iSGCs and pSGCs were harvested and dissociated to single cells with 0.25% trypsin–EDTA (Solarbio), then suspended in D-Hank’s without calcium loaded with Ca^2+^ indicator dyes (C3015, calcium orange™, AM, Invitrogen) for 30 min. Cells were then washed three times in D-Hank’s without calcium and resuspended in 200 µl CaCl_2_ solution (2 mmol/L, Sigma-Aldrich) containing acetylcholine chloride (50 µmol/L, Sigma-Aldrich) for immediate analysis and flow cytometry quantification.

### Animals and transplantation

The animal experiment was performed according to protocols approved by the Ethics Committee at the Fourth Medical Center of PLA General Hospital, in accordance with Institutional Animal Care and Use Committee (IACUC) guidelines [19]. BALB/c nude mice were randomly divided into a normal group (*n* = 60), modified SGM treatment group (*n* = 60) and iSGC transplantation group (*n* = 60). For the establishment of a mouse burn model, athymic BALB/c nude mice (female, 8 weeks old) were purchased from SiBeiFu (Beijing, China). For the establishment of a mouse model with damaged SGs in the dermis, after anesthesia with pentobarbital (100 mg/kg) and routine disinfection, the paw pads of the mice were placed in contact with a metal scalding instrument for 5 s at 65℃, then dipped into ice water for 3 s to remove retained heat [20]. On day 3 and day 5 after thermal injury, iSGCs [5 × 10^5^ cells in 50 μl mSGM] were collected and injected into the paw pads of recipient mice. Simultaneously, 50 μl mSGM was intradermally injected in the scalded paw pads of the vehicle groups. After engraftment, mice were monitored daily and sacrificed on day 21 if the burn wounds of the paw pads had healed [20].

### Sweat tests

The paw pads were first coated with a 2% (W/V) iodine/ethanol solution, followed by starch. After drying, 50 µl acetylcholine chloride (100 µmol/L, Sigma-Aldrich) was injected subcutaneously into the paws of mice.

### Statistical analysis

All values are presented as the means ± standard deviation (SD) calculated from the average of at least three biological replicates unless otherwise specified. Statistical analysis was performed in GraphPad Prism 8.0. Comparisons between two groups were analyzed with Student’s *t* test. *P* values < 0.05 were considered statistically significant.

## Results

### EDA alone is not sufficient for reprogramming HDFs to iSGCs

**Fig. 1:**
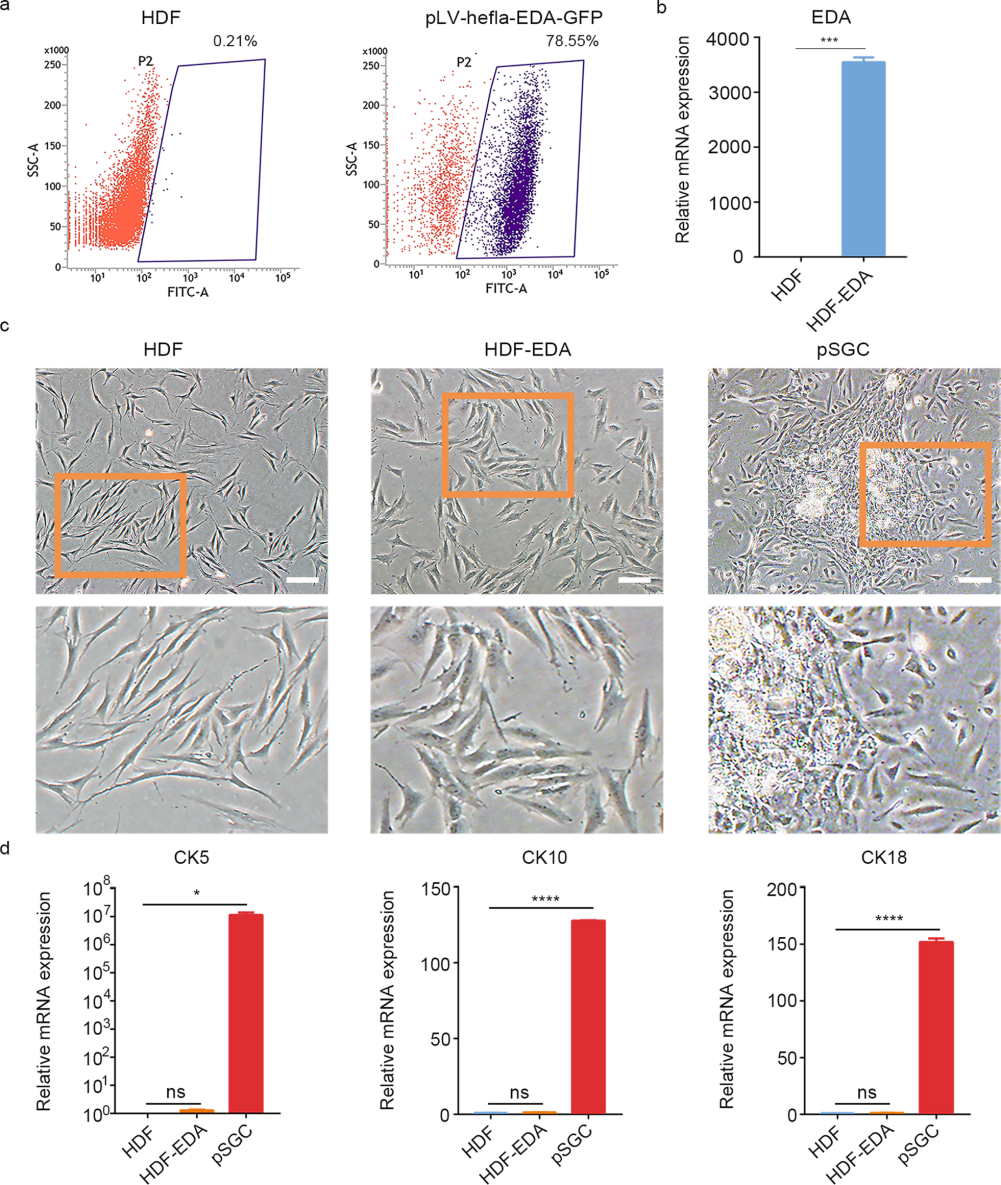
EDA alone is not sufficient for reprogramming HDFs into iSGCs. **a** Flow cytometry quantification of GFP^+^ cells. Fluorescence intensity of GFP^+^ cells enriched by puromycin. Human dermal fibroblasts were transfected with EDA on day 15. **b** Confirmation of successful transfection with EDA by qPCR analysis. **c** Phase contrast images showing the morphological difference between HDF, HDF-EDA and pSGCs. Scale bar = 200 μm. Illustrations, higher magnification of the boxed areas. **d** qPCR analysis of transcriptional expression of CK5, CK10 and CK18 in HDF, HDF-EDA and pSGC. The genes showing significant differences in qPCR array assay were presented. *n* = 3. Data were expressed as mean ± SD and analyzed by two-tailed *t*-tests, ^*^P < 0.05, ^***^P < 0.001, ^****^P < 0.0001, ns not significant, EDA ectodermal dysplasia antigen, HDF human dermal fibroblasts, CK5 cytokeratin 5, CK10 cytokeratin 10, CK18 cytokeratin 18, pSGC primary sweat gland cell, iSGC induced sweat gland-like cell

To direct HDFs toward the SG lineage, we introduced the single factor EDA into HDFs. The greatest number of GFP^+^ cells was produced when the transfected cells were enriched with 0.4 µg/ml puromycin for 15 d (HDF-EDA), with GFP^+^ cells comprising 78.55% of all cells (Fig. 1a). Transgene expression in the HDF-EDA cells compared with the HDF control was confirmed by qPCR (*P* < 0.001; Fig. 1b). Although EDA overexpression induced morphological changes, the prominent cell bodies differed from the typical SG cell morphology (Fig. 1c). The qPCR results further indicated that the SG cell associated-markers CK5, CK10 and CK18 did not increase significantly in HDF-EDA cells on day 15 after culture in fibroblast medium (Fig. 1d), thus indicating that the single factor EDA was not sufficient to enable HDFs to acquire the phenotype of SG cells.

### Generation of iSGCs from HDFs by using EDA and SGM

**Fig. 2:**
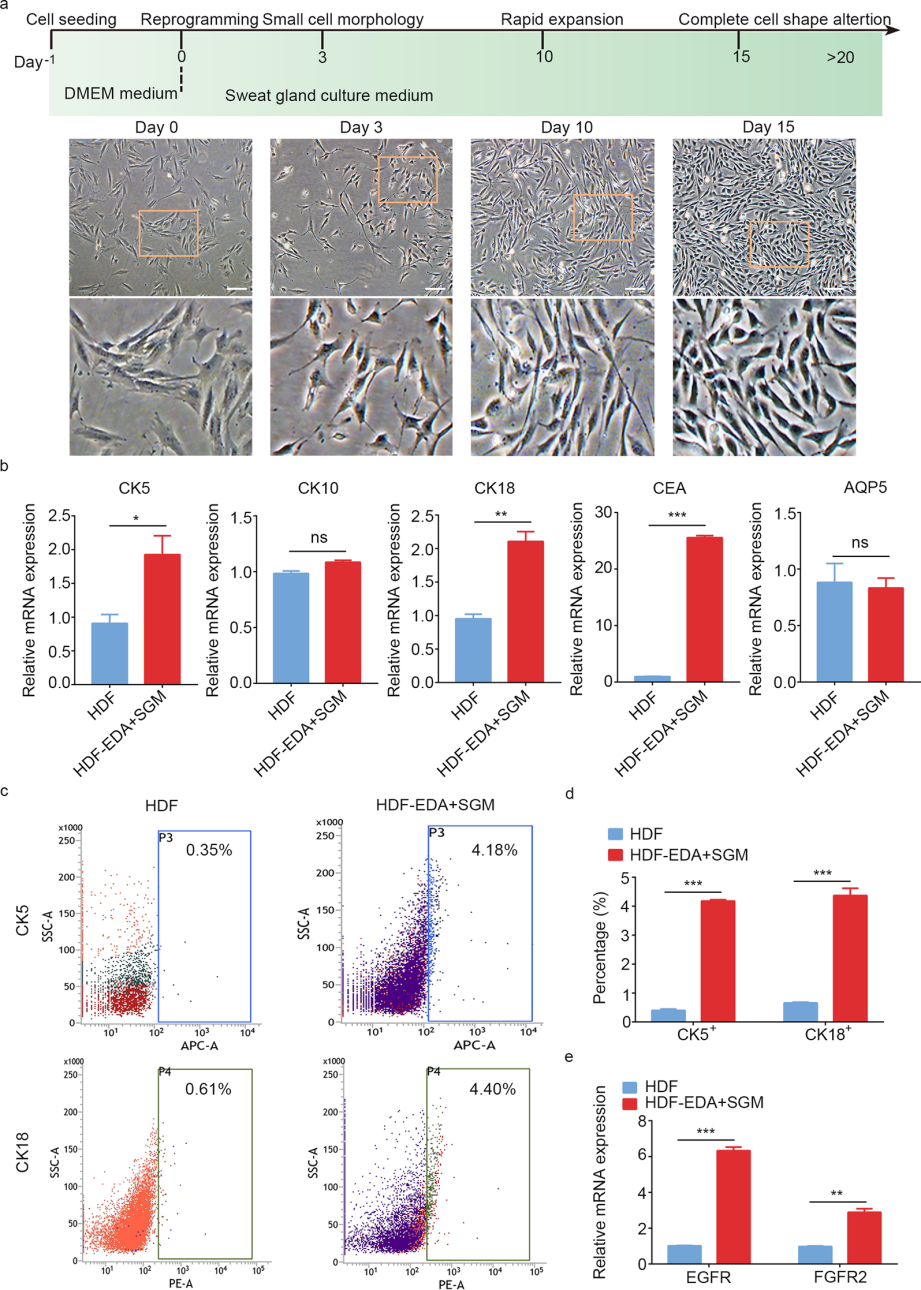
Acquisition of SG cells phenotype by forced transgenic expression of EDA and SGM culture. **a** Phase contrast images showing the morphological changes of HF-EDA in SGM on day 0, 3, 10 and 15. Scale bar = 200 μm. Illustrations, higher magnification of the boxed areas. **b** qPCR analysis of transcriptional expression of CK5, CK10, CK18, CEA and AQP5 in HDF and HDF-EDA cultured in SGM after 15 d of induction. **c, d** Flow cytometry quantification of CK5 positive cells and CK18 positive cells of HDF-EDA in SGM. **e** qPCR analysis of EGFR and FGFR2 mRNA levels in HDF-EDA cultured in SGM. *n* = 3. Data were expressed as mean ± SD and analyzed by two-tailed *t*-tests, ^*^P < 0.05, ^**^P < 0.01, ^***^P < 0.001, ns not significant, SG sweat gland, SGM sweat gland culture medium, EDA ectodermal dysplasia antigen, CK5 cytokeratin 5, CK10 cytokeratin 10, CK18 cytokeratin 18, CEA carcino-embryonic antigen, AQP5 aquaporin 5, HDF human dermal fibroblasts, EGFR epidermal growth factor receptor, FGFR2 fibroblast growth factor receptor 2

Given these results, we switched the HDF-EDA cells from fibroblast medium to SGM containing epidermal growth factor and basic fibroblast growth factor. On day 3 after SGM treatment, HDF-EDA cells with small cell morphology gradually emerged. On day 10, SGM cultures led to a rapid expansion in the number of HDF-EDA cells. After prolonged exposure to SGM until day 15–20, the epidermal-like morphology of most induced cells began to appear (Fig. 2a). RNA expression profiling by qPCR revealed significantly increased levels of the key SG genes CK5 (*P* < 0.05), CK18 (*P* < 0.01) and CEA (*P* < 0.001), but not the SG duct cell marker CK10 and luminal functional marker AQP5, in HDF-EDA cells treated with SGM (Fig. 2b). FACS analysis demonstrated that, after SGM treatment, approximately (4.18 ± 0.04)% of the HDF-EDA cells expressed CK5 (*P* < 0.001), and (4.36 ± 0.25)% expressed CK18 (*P* < 0.001) (Fig. 2c, d). Immunofluorescence marker expression analysis of HDF-EDA cells in SGM culture indicated the expression of myoepithelial cell-associated proteins CK5 and SMA (Additional file 2: Fig. S1). We then performed qPCR to examine the transcriptional changes in epidermal growth factor receptor (EGFR) and fibroblast growth factor receptor 2 (FGFR2) in SGM culture. As expected, significantly increased mRNA levels of EGFR (*P* < 0.001) and FGFR2 (*P* < 0.01) were observed in HDF-EDA cells treated with SGM (Fig. 2e). However, although long-term exposure to SGM upregulated CK5, CK18 and CEA transcription in HDF-EDA cells, only a small proportion of the reprogrammed cells could be induced to express CK5 and CK18, thus indicating a partial and inefficient conversion, and suggesting that additional treatments were required to increase the conversion efficiency of iSGCs.

### Small molecules facilitate iSGCs generation from HDFs

**Fig. 3:**
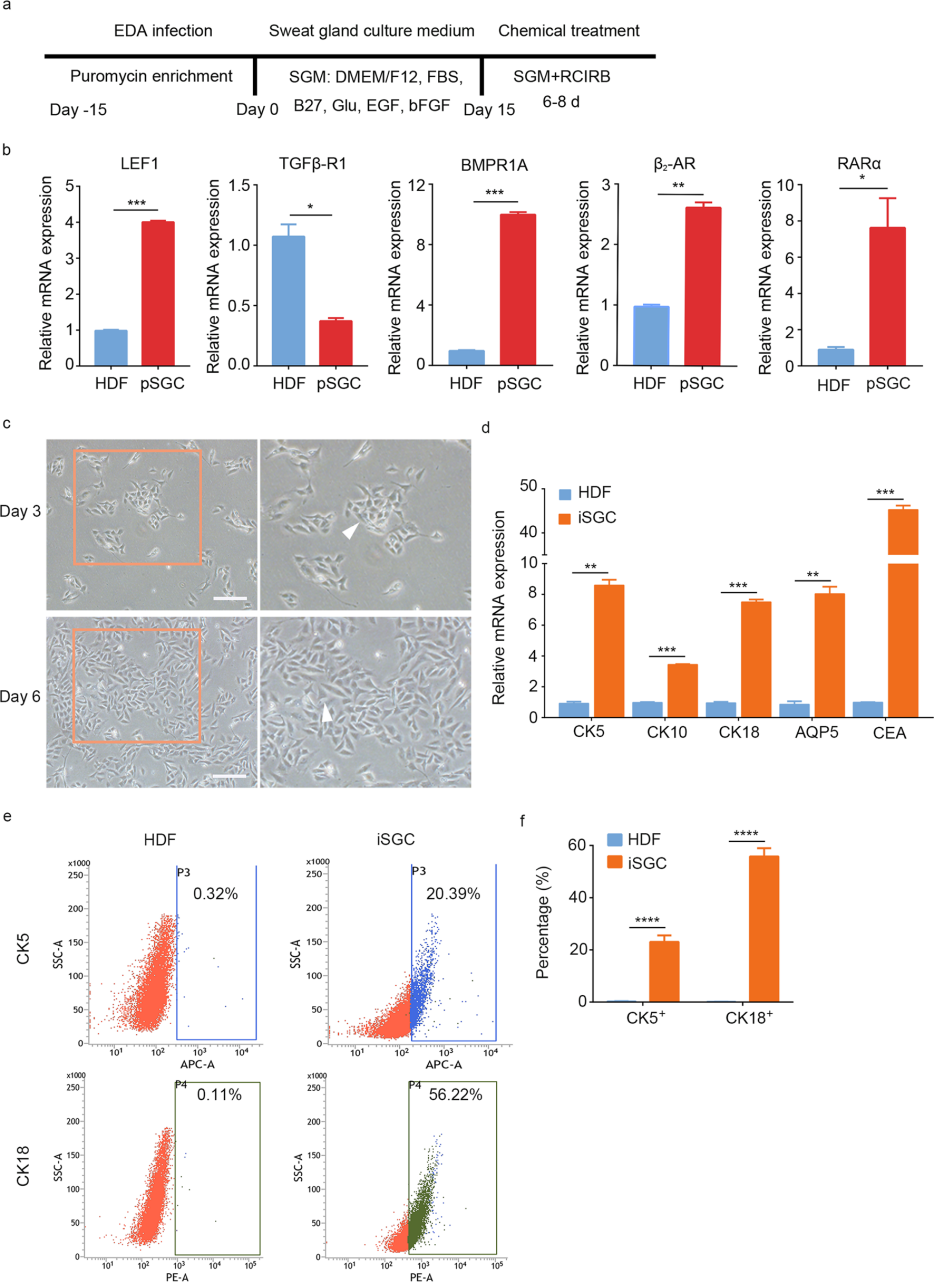
Conversion of HDF into iSGC with small molecules. **a** Scheme of reprogramming strategy procedure. HDF was transfected with EDA and plated in fibroblasts medium, then cells were transferred into SGM for 15–20 d, and eventually HDF-EDA in SGM were further transferred into mSGM for 6–8 d. **b** qPCR analysis of transcriptional expression of LEF1, TGFβ-R1, BMPR1A, β_2_-AR and RARα in HDF and pSGC; The genes showing significant differences were observed. **c** Phase contrast images showing the morphological changes of iSGC in mSGM on day 3 and 6. Scale bar = 200 μm. Illustrations, higher magnification of the boxed areas. **d** qPCR analysis of transcriptional expression of CK5, CK10, CK18, AQP5 and CEA in HDF and iSGC after 6—8 d of induction. **e, f** Flow cytometry quantification of CK5 positive cells and CK18 positive cells of iSGC. *n* = 3. Data were expressed as mean ± SD and analyzed by two-tailed *t*-tests, ^*^P < 0.05, ^**^P < 0.01, ^***^P < 0.001, ^****^P < 0.0001. EDA ectodermal dysplasia antigen, SGM sweat gland culture medium, EGF epidermal growth factor, bFGF basic fibroblast growth factor, RCIRB (Repsox, CHIR99021, Isoproterenol, Retinoic acid, BMP4), HDF human dermal fibroblasts, pSGC primary sweat gland cell, LEF1 lymphoid enhancer-binding factor 1, TGFβ-R1 TGF-β type 1 receptor, BMPR1A bone morphogenetic protein receptor 1A, β_2_-AR β_2_-adrenergic receptor, RARα retinoic acid receptor α, iSGC induced sweat gland-like cell, mSGM modified sweat gland culture medium, CK5 cytokeratin 5, CK10 cytokeratin 10, CK18 cytokeratin 18, AQP5 aquaporin 5, CEA carcino-embryonic antigen

We hypothesized that the cells induced with a combination of small molecules modulating SG-developmental signaling would promote the SG cell transcriptional program in HDF-EDA cells. In light of the above results, we designed the strategy in Fig. 3a to systemically reprogram HDFs to iSGCs by combining small molecule treatment, forced transgenic expression of EDA and SGM culture. The qPCR results demonstrated significantly lower mRNA levels of TGFβ-R1 (*P* < 0.05) and higher mRNA levels of lymphoid enhancer-binding factor 1 (LEF1) (*P* < 0.001), bone morphogenetic protein receptor 1A (BMPR1A) (*P* < 0.001), β_2_-adrenergic receptor (β_2_-AR) (*P* < 0.01) and retinoic acid receptor α (RARα) (*P* < 0.05) in SG cells than HDFs (Fig. 3b). Therefore, Repsox (a TGFβ-R1 inhibitor), CHIR99021 (a WNT signaling agonist), isoproterenol (a β_2_-AR agonist), BMP4 and RA (a RARα agonist) were combined (treatment denoted RCIBR) in SGM for iSGCs induction; this medium was denoted mSGM. At approximately 6–8 d after treatment with mSGM, the iSGCs exhibited SG cell-like morphology (Fig. 3c). RNA expression profiling by qPCR revealed significantly increased levels of CK5 (*P* < 0.01), CK18 (*P* < 0.001) and CEA (*P* < 0.001), and the duct cell marker CK10 (*P* < 0.001) and luminal marker AQP5 (*P* < 0.01) were also detected (Fig. 3d). FACS analysis further indicated that approximately (23.05 ± 2.49)% of iSGCs were CK5 positive (*P* < 0.0001), and (55.79 ± 3.18)% of iSGCs were CK18 positive (*P* < 0.0001; Fig. 3e, f). These results demonstrated that small molecules can promote the acquisition of SG cell properties by fibroblasts and greatly improve reprogramming efficiency.

### Functional analysis of iSGCs

**Fig. 4:**
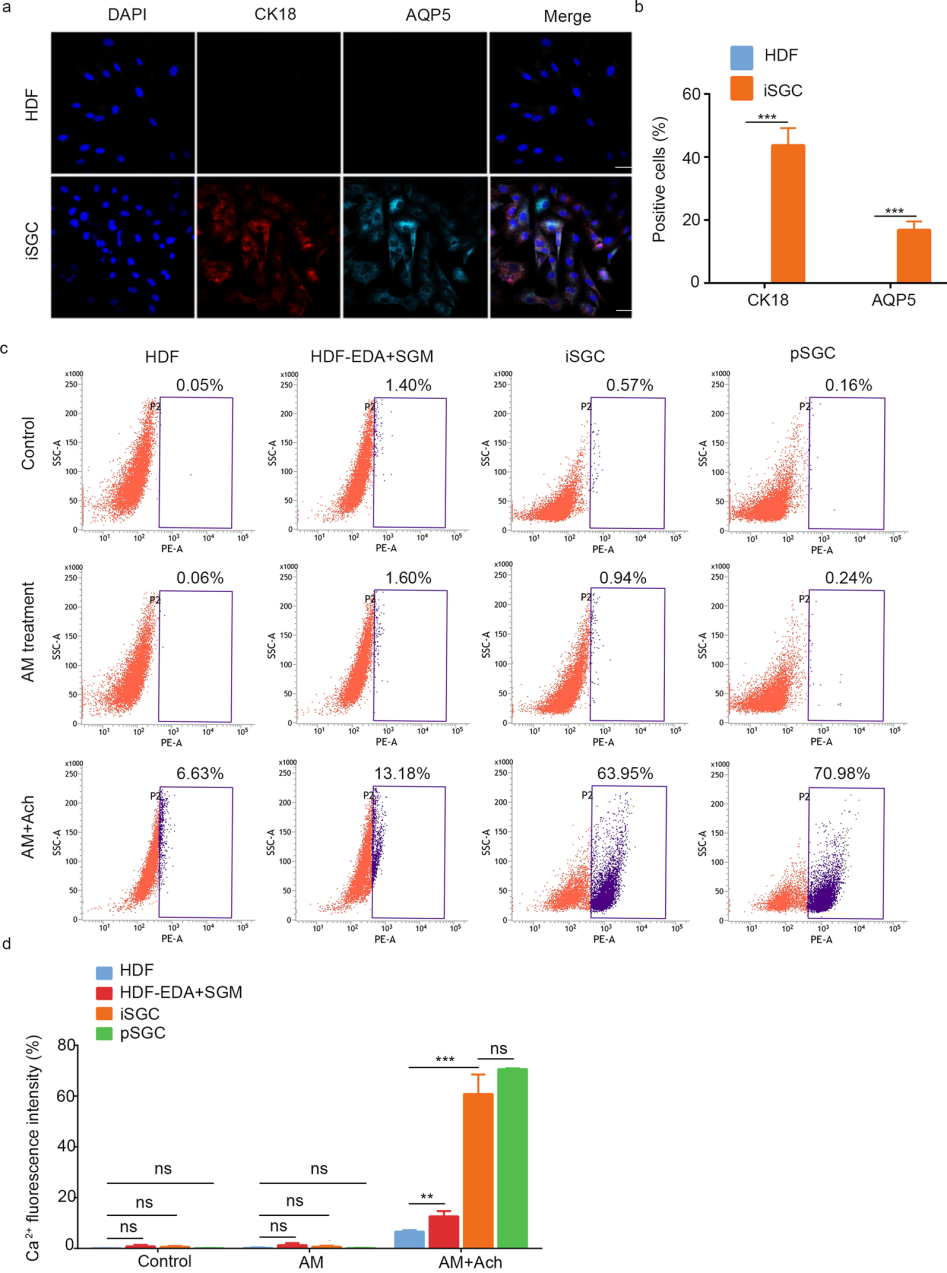
Functional analysis of iSGC. **a** Representative immunofluorescence of CK18^+^ and AQP5^+^ in HDF and iSGC. Scale bar = 50 μm. **b** Percentages of CK18^+^ and AQP5^+^ cells in HDF and iSGC calculated according to the immunostaining. Quantification was done with 5 randomly selected microscopy fields from each of the 3 independent experiments. **c** Calcium activity analysis was used to assess the reactivity to acetylcholine. **d** The data presented the intracellular free Ca^2+^ intensity of iSGCs was higher than HF-EDA in SGM and similar to that of the pSGC, (60.79 ± 7.71)%, (12.65 ± 2.07)% and (70.59 ± 0.34)%, respectively. *n* = 3. Data were expressed as mean ± SD and analyzed by two-tailed *t*-tests, ^**^P < 0.01, ^***^P < 0.001*,* ns not significant, HDF human dermal fibroblasts, iSGC induced sweat gland-like cell, CK18 cytokeratin 18, AQP5 aquaporin 5, EDA ectodermal dysplasia antigen, SGM sweat gland culture medium, pSGC primary sweat gland cell, ns not significant

Having validated the expression of SG-associated markers in the iSGCs, we next evaluated their subpopulation composition by immunostaining for the luminal markers AQP5 and CK18. Immunofluorescence results indicated a significantly greater AQP5/CK18-positive fraction in the iSGCs than the HDF controls (Fig. 4a, b). The ability to respond appropriately to hormones and transmitters of the autonomic nervous system has been reported to be one of the most critical functional characteristics of native SG cells. We therefore sought to determine how HDF-derived induced SG cells responded to muscarinic stimulation. We measured intracellular Ca^2+^ signals by using an orange fluorescent calcium indicator. As shown in Fig. 4c, d, the addition of the muscarinic agonist acetylcholine chloride (Ach) significantly increased spontaneous calcium transients. Compared with that in HDFs (6.64 ± 0.57)%, the intracellular free Ca^2+^ intensity of HDF-EDA treated with SGM was significantly higher [(12.65 ± 2.07)%; *P* < 0.01] but was still much lower than that in pSGCs (70.59 ± 0.34)%. After 6–8 d of mSGM incubation, the intracellular free Ca^2+^ intensity in iSGC was approximately (60.79 ± 7.71)%, a value comparable to that of pSGCs. These results indicated that the iSGCs were functionally similar to normal SG cells.

### iSGC engraftment reconstitutes damaged skin with fully restored SG functions

**Fig. 5:**
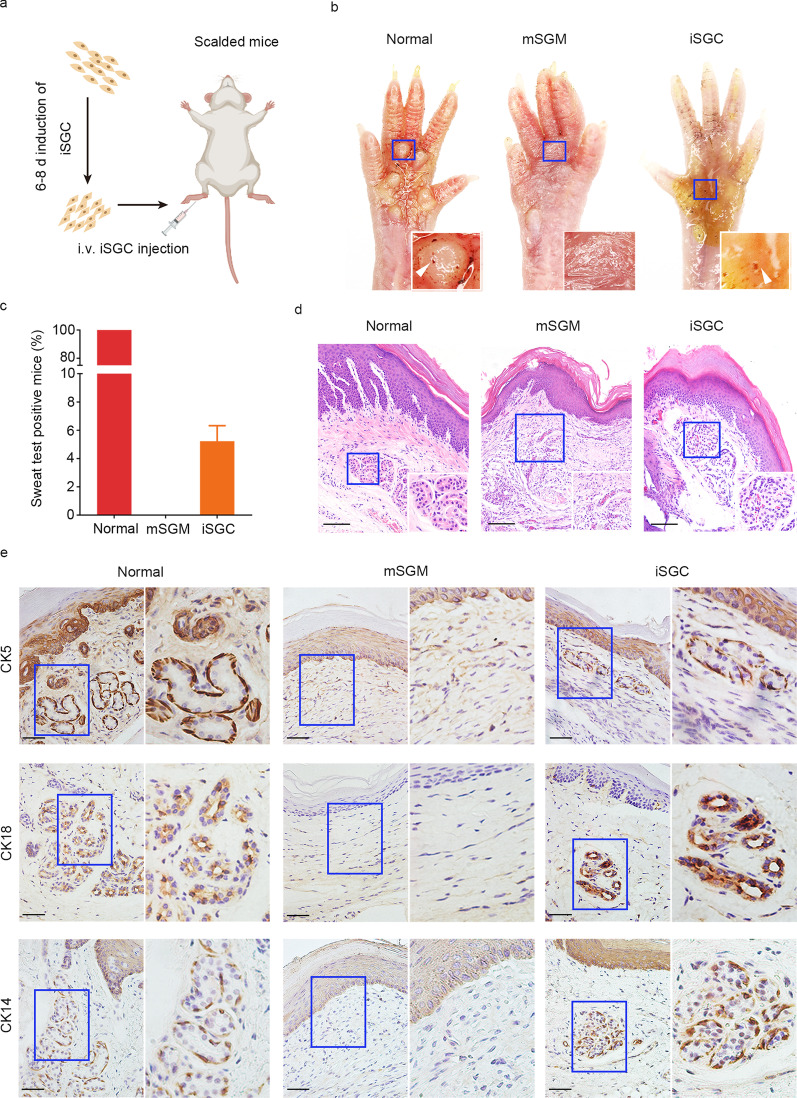
Engraftment of iSGC functionally restored SG. **a** Schematic diagram representing the experimental procedure. **b** Starch-iodine sweat tests on paw skin of thermal-injured mice showed that paw pads of mSGM-treated mice (*n* = 60), iSGC-treated mice (*n* = 60) and normal mice (*n* = 60) responded by displaying indigo-black dots on day 21 after transplantation. **c** The positive rate of sweat test of the iSGC-treated group, (5.2 ± 1.1)% of the recipient mice (*n* = 60). **d** H&E staining was conducted to visualize normal group and mSGM- and iSGC-treated wounds on day 21 post-injury. Emerging glandular structures were seen in the dermis of iSGC-treated mice and normal mice. Scale bar = 50 μm. Illustrations, higher magnification of the boxed areas. **e** The SG markers CK5, CK18, and CK14 were assessed by immunohistochemical analysis to examine the SG formation. The results showed that, like natural paw shin, the iSGC-treated group could form SG-like structures with positive staining for the SG markers, while no SG regeneration was observed in mSGM-treated group. Scale bars = 50 μm. Illustrations, higher magnification of the boxed areas. iSGC induced sweat gland-like cell, SG sweat gland, mSGM modified sweat gland culture medium, CK5 cytokeratin 5, CK18 cytokeratin 18, CK14 cytokeratin 14

To address whether the generated iSGCs were able to functionally restore SGs, we subcutaneously injected iSGCs into the scalded mouse paw pads (Fig. 5a). After 21 d, starch-iodine sweat tests on the paw pads indicated that iSGC-treated mice responded to the assay by displaying indigo-black dots (Fig. 5b). Approximately (5.2 ± 1.1)% of the mice were sweat test positive on day 21 after treatment with iSGCs (Fig. 5c). The histological analysis results indicated that regenerated SG structures were present in iSGC-treated mice (Fig. 5d). We additionally observed the expression of the SG markers CK5, CK18 and CK14 was similar to that in the normal group (Fig. 5d). As expected, the skin tissues of the mSGM group showed only damaged structures without the SG components and corresponding markers (Fig. 5d, e). Additionally, no tumors were seen in iSGC-treated mice, whereas xenograft tumors were observed in the MGC-803 gastric cell line group (Additional file 2: Fig. S2). Collectively, these results indicated that iSGCs promote SG regeneration structurally and functionally in vivo.

## Discussion

In the present study, we first investigated the key factors governing the cell-fate transition of fibroblasts into SG cells by exploring the kinetics of the direct conversion process. We found that the single factor EDA successfully reprogrammed human fibroblasts into induced SG cells when it was combined with small-molecule cocktails. Systematic characterization indicated that the iSGCs exhibited several typical features of SG cells, displaying expression of duct- and luminal-specific markers, and elevated muscarinic reactivity. Notably, we identified small molecules enhancing the efficiency of SG cell reprogramming. The iSGCs generated through our method acquired the biochemical and functional characteristics of native SG cells in vitro; importantly, subcutaneous transplantation of these iSGCs restored and regenerated SG.

Regeneration of skin appendages is key to the functional repair of large-scale skin defects. Wound-resident cells are present but insufficient to promote de novo SG morphogenesis [3]. Direct lineage reprogramming can achieve direct induction of functional cell types from one lineage to another, without cells passing through an intermediate pluripotent stage [21]. This technique has been successfully used to regenerate various cells, such as cardiomyocytes and hepatocytes [22, 23]. Therefore, direct lineage reprogramming may provide an alternative method to produce SG cells. Fibroblasts are the main cells involved in skin injury repair. Reprogramming fibroblasts to SG cells in vitro will be important for in situ regeneration of SGs in the future. Fibroblasts are also widely available and easy to access and culture. Therefore, we sought to direct the fate of fibroblasts to SG cells. Recently, a direct conversion technology mediated by lineage-specific factors has been developed, and previous studies have demonstrated that fibroblasts can be directly converted to hepatocyte cells [24], inner ear hair cells [25], myogenic progenitor cells [26] and cardiomyocytes [27] through treatment with defined factors. Accumulating evidence suggests that EDA plays an essential role in SG morphogenesis [28, 29], and defective development of SG in hypohidrotic ectodermal dysplasia can be rescued by EDA administration [30]. Our previous work has also demonstrated that CRISPR/dCas9-effector targeting of the ectodysplasin promoter effectively induces the reprogramming of human bone marrow-derived mesenchymal stem cells and epidermal cells to SG cells [4, 5]. These transfected cells not only express SG related markers (CK5, CK10, CK18, CEA, CK7, CK14 and CK19) but also contribute to SG reconstruction in vivo [4, 5]. Therefore, EDA could reasonably be used to induce SG fate. In the present study, we successfully overexpressed EDA in HDFs and found that this single factor together with SGM containing growth factors enabled HDF-EDA cells to preliminarily acquire a partial SG cell phenotype, with the expression of SG markers (CEA, CK5 and CK18). However, the induction efficiency was relatively low: only (4.18 ± 0.04)% of HDF-EDA cells treated with SGM expressed CK5, and (4.36 ± 0.25)% expressed CK18. Furthermore, a ductal marker (CK10) and luminal marker (AQP5) were not detectable, and the muscarinic reactivity substantially differed from that of pSGCs [(12.65 ± 2.07)% vs. (70.59 ± 0.34)%]. Therefore, further exploration of additional factors that may boost the activation of the SG program was necessary.

Developmentally, SG fate is highly susceptible to WNT/β-catenin/lymphoid/LEF1 signaling pathways. WNT5A and WNT10A cause accumulation of nuclear β-catenin in mesenchymal components during cutaneous appendage morphogenesis [31]. WNT10A mutations account for 16% of hypohidrotic ectodermal dysplasias [32]. In addition, the WNT signaling pathway and the EDA/EDAR axis together guide SwG morphogenesis. For example, LEF1 binds the 1.6-kb DNA sequence upstream of the EDA transcriptional initiation site and further activates the EDA/EDAR signaling pathway [33]. The bone morphogenetic protein (BMP) signaling pathway also mediates a switch to SG fate in skin. A previous study has demonstrated that BMPR1A transcripts are markedly elevated in SG-permissive epidermis, and the activation of BMP signaling determines SG formation, whereas HF morphogenesis occurs in mouse paws if BMP signaling is suppressed [34]. In addition, cocultured epidermal stem cells with embryonic paw pad tissue exhibit a glandular structure, and BMP4 concentrations have been detected in the medium; moreover, a BMPR1A inhibitor effectively blocks differentiation of these cells [34], thus further suggesting the role of BMP4 activation in SG regeneration. SG is a peripheral neuroendocrine-control organ. The activation of β-adrenoceptors opens Ca^2+^ channels, thereby elevating intracellular Ca^2+^ concentration and eventually increasing sweat secretion. We speculated that neuroendocrine signaling might further drive SG fate, and also observed an expression difference in β_2_-AR between fibroblasts and pSGCs. Fibroblasts originate mainly from the mesoderm, and SGs are ectoderm organ. RA not only plays an important role in initiating ectoderm development [35, 36], but also participates in exocrine gland formation [37, 38]. Moreover, the cooperation of BMP4 with RA promotes the expression of CK18, CK5 and CK14 (the obligate partner of CK5) [39]. TGFβ signaling mediates mesoderm development, and inhibition of TGFβ signaling is necessary for the fate switch from fibroblasts or astrocytes to other cells [40, 41]. The activation of WNT signaling and BMP signaling and the inhibition of TGFβ signaling have been found to increase CK18, CK14 and CK10 [42]. Interestingly, our results also confirmed the differences in the above signaling pathways between fibroblasts and pSGCs, thus suggesting that targeted regulation of these pathways may enhance the efficiency of SG reprogramming. Therefore, we added Repsox (a TGFβ signaling inhibitor), CHIR99021 (a WNT signaling activator), isoproterenol, RA and BMP4 to SGM to produce mSGM. After further induction for 6–8 d, EDA, in cooperation with small molecules, enabled the direct conversion of fibroblasts into iSGCs with high yield. Not only did the expression of CK5, CK18 and CEA further increase, but CK10 and AQP5 were upregulated. Among the induced cells, (23.05 ± 2.49)% were CK5 positive, and (55.79 ± 3.18)% were CK18 positive. The reactivity to acetylcholine was greatly improved and was highly similar to that of pSGCs [(60.79 ± 7.71)% vs. (70.59 ± 0.34)%, ns].

In vivo transplantation experiments showed that HDF-derived iSGCs promoted SG regeneration, and the emerging SGs in the iSGC transplantation group exhibited structural and phenotypical characteristics similar to those in the normal group: both secretory cells and luminal epithelial layers were present, and responsiveness to acetylcholine was observed after engraftment to mouse paw skin with second-degree burns. Collectively, the iSGCs derived from HDF-EDA in mSGM may serve as a cell source to regenerate SGs.

Limitations of this study included the use of an immunodeficient mouse model and gene editing mediated reprogramming. The immunodeficient mouse model does not fully simulate the human microenvironment in vivo. Immunologically competent mice have an immune environment similar to that in humans, thus serving as a good model for preclinical experiments. We also expect that iSGCs generated from this study could facilitate SG regeneration in an immunologically competent mouse model. However, much research remains to be performed on iSGCs engraftment in immunologically competent mice, such as to determine how to decrease immune rejection and ensure the in vivo survival of iSGCs. Herein, we achieved the conversion of HDFs to iSGCs via gene editing. In the future, methods of small molecule-mediated chemical reprogramming alone without EDA overexpression to obtain HDF-derived iSGCs must be further explored.

## Conclusions

In summary, we established a direct reprogramming strategy for the induction of SG cell regeneration from HDFs in vitro. The genetic approach drives the fate of desired functional cells during reprogramming, and combination with a chemical approach further promotes cell-fate conversion. This strategy enables the efficient conversion of HDFs to iSGCs in vitro and permits the generation of desired functional SGs for in vivo treatment.

## Supplementary Information

**Additional file 1:** **Table S1** Primer sequences used in the study.

**Additional file 2:** **Fig. S1**. Generation of iSGC from HDF via EDA activation together with SGM culture. Immunofluorescence assay of expression patterns of HDF-EDA cells in SGM culture, mainly expressing myoepithelial cell-associated proteins CK5 and α-SMA. Scale bar = 50 μm. iSGC induced sweat gland-like cell, HDF human dermal fibroblasts, EDA ectodermal dysplasia antigen, SGM sweat gland culture medium, CK5 cytokeratin 5, α-SMA α-smooth muscle actin. **Fig. S2**. In vivo safety assessment of iSGC. MCG-803 gastric cell lines were used as positive controls. Cells were collected and injected subcutaneously at 1 × 107 cells per site. On day 25 after implantation, no tumors were seen in iSGC-treated mice, whereas xenograft tumors were observed in MGC803 group. n = 4. iSGC induced sweat gland-like cell.

## Data Availability

The data and materials used in the current study are all available from the corresponding author upon reasonable request.

## Abbreviations

Ach: Acetylcholine chloride
AQP5: Aquaporin 5
BMP: Bone morphogenetic protein
BMPR1A: Bone morphogenetic protein receptor 1A
CEA: Carcino-embryonic antigen
CK10: Cytokeratin 10
CK14: Cytokeratin 14
CK18: Cytokeratin 18
CK19: Cytokeratin 19
CK5: Cytokeratin 5
CK7: Cytokeratin 7
EDA: Ectodermal dysplasia antigen
EGF: Epidermal growth factor
EGFR: Epidermal growth factor receptor
FGFR2: Fibroblast growth factor receptor 2
HDF: Human dermal fibroblasts
iSGCs: Induced sweat gland-like cells
LEF1: Lymphoid enhancer-binding factor 1
mSGM: Modified sweat gland culture medium
ns: Not significant
PBS: Phosphate-buffered saline
pSGCs: Primary sweat gland cells
qPCR: Quantitative PCR
RA: Retinoic acid
RARα: Retinoic acid receptor α
SG: Sweat gland
SGM: Sweat gland culture medium
TGFβ-R1: TGF-β type 1 receptor
β_2_-AR: β_2_-Adrenergic receptor
α-SMA: α-Smooth muscle actin
