# Supplementary material for: Small molecules facilitate single factor-mediated sweat gland cell reprogramming
Source: Mil Med Res. 2022 Mar 29;9:13. doi: 10.1186/s40779-022-00372-5 (PMC8962256; doi:10.1186/s40779-022-00372-5)
Supplement: Supplementary file 1 — Additional file 1: Table S1 Primer sequences used in the study. [file 40779_2022_372_MOESM1_ESM.pdf]

Table S1 Primer sequences used in the study

Genes	Forward primer	Reverse primer
CK5	GGAGAAGGAGTTGGACCAGTCAAC	CTACCTCCGGCAAGACCTCCAC
CK10	ATGTCTGTTCGATACAGCTCAAG	CTCCACCAAGGGAGCCTTTG
CK18	GTTGACCGTGGAGGTAGATGC	GAGCCAGCTCGTCATATTGGG
CEA	TAAGTGTTGACCACAGCGACCC	GTTCCCATCAATCAGCCAAGAA
AQP5	CGGGCTTTCTTCTACGTGG	GCTGGAAGGTCAGAATCAGCTC
β_2-AR	TTGCTGGCACCCAATAGAAGC	CAGACGCTCGAACTTGGCA
TGFβ-R1	ACGGCGTTACAGTGTTTCTG	GCACATACAAACGGCCTATCTC
LEF1	AGAACACCCCGATGACGGA	GGCATCATTATGTACCCGGAAT
BMPR1A	AGATGACCAGGGAGAAACCAC	CAACATTCTATTGTCCGGCGTA
FGFR2	AGCACCATACTGGACCAACAC	GGCAGCGAAACTTGACAGTG
EGFR	TTGCCGCAAAGTGTGTAACG	GTCACCCCTAAATGCCACCG
RARα	AAGCCCGAGTGCTCTGAGA	TTCGTAGTGTATTTGCCCAGC
EDA	GGACGGCACCTACTTCATCT	TGTAGTTGGTCTTGCCCGTC
β-ACTIN	CATGTACGTTGCTATCCAGGC	CTCCTTAATGTCACGCACGAT

CK5 cytokeratin 5, *CK10* cytokeratin 10, *CK18* cytokeratin 18, *CEA* carcino-embryonic antigen, *AQP5* aquaporin 5, *β_2-AR* β_2-adrenergic receptor, *TGFβ -R1* TGF-β type 1 receptor, *LEF1* lymphoid enhancer-binding factor 1, *BMPR1A* bone morphogenetic protein receptor 1A, *FGFR2* fibroblast growth factor receptor 2, *EGFR* epidermal growth factor receptor, *RARα* retinoic acid receptor α, *EDA* ectodermal dysplasia antigen
