# Supplementary material for: Small molecules facilitate single factor-mediated sweat gland cell reprogramming
Source: Mil Med Res. 2022 Mar 29;9:13. doi: 10.1186/s40779-022-00372-5 (PMC8962256; doi:10.1186/s40779-022-00372-5)
Supplement: Supplementary file 2 — Additional file 2: Fig. S1. Generation of iSGC from HDF via EDA activation together with SGM culture. Immunofluorescence assay of expression patterns of HDF-EDA cells in SGM culture, mainly expressing myoepithelial cell-associated proteins CK5 and α-SMA. Scale bar = 50 μm. iSGC induced sweat gland-like cell, HDF human dermal fibroblasts, EDA ectodermal dysplasia antigen, SGM sweat gland culture medium, CK5 cytokeratin 5, α-SMA α-smooth muscle actin. Fig. S2. In vivo safety assessment of iSGC. MCG-803 gastric cell lines were used as positive controls. Cells were collected and injected subcutaneously at 1 × 10^7^ cells per site. On day 25 after implantation, no tumors were seen in iSGC-treated mice, whereas xenograft tumors were observed in MGC803 group. n = 4. iSGC induced sweat gland-like cell. [file 40779_2022_372_MOESM2_ESM.pdf]

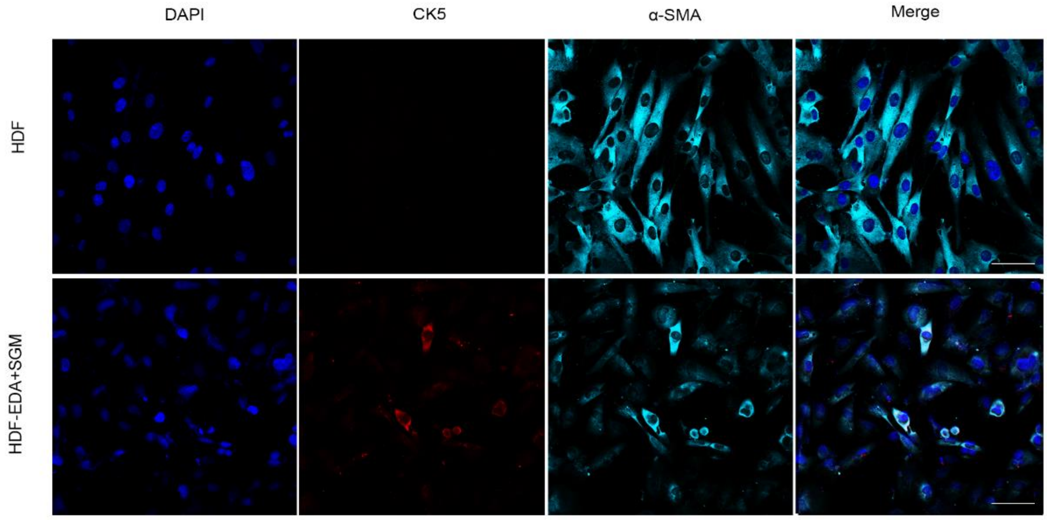

Fig. S1 Generation of iSGC from HDF via EDA activation together with SGM culture. Immunofluorescence assay of expression patterns of HDF-EDA cells in SGM culture, mainly expressing myoepithelial cell-associated proteins CK5 and α-SMA. Scale bar = 50 μm. iSGC induced sweat gland-like cell, HDF human dermal fibroblasts, EDA ectodermal dysplasia antigen, SGM sweat gland culture medium, CK5 cytokeratin 5, α-SMA α-smooth muscle actin

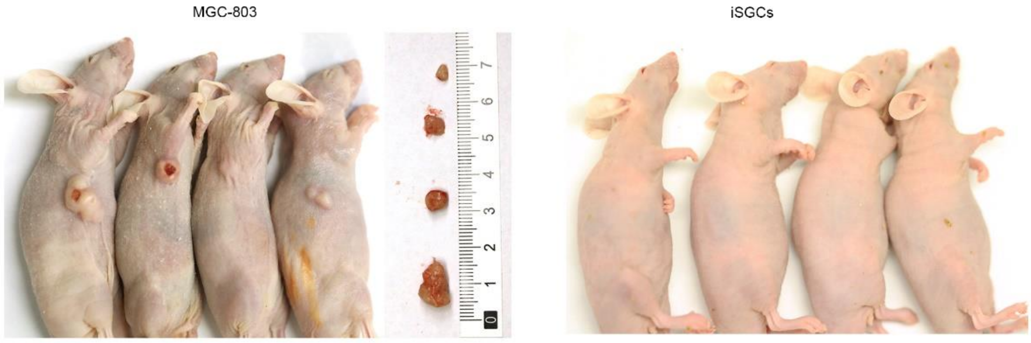

Fig. S2 In vivo safety assessment of iSGC. MCG-803 gastric cell lines were used as positive controls. Cells were collected and injected subcutaneously at 1×10^7 cells per site. On day 25 after implantation, no tumors were seen in iSGC-treated mice, whereas xenograft tumors were observed in MGC803 group. n = 4. iSGC induced sweat gland-like cell
